# Genomic Signatures of Selection Associated With Litter Size Trait in Jining Gray Goat

**DOI:** 10.3389/fgene.2020.00286

**Published:** 2020-03-26

**Authors:** Jun-Jie Wang, Teng Zhang, Qiu-Ming Chen, Rui-Qian Zhang, Lan Li, Shun-Feng Cheng, Wei Shen, Chu-Zhao Lei

**Affiliations:** ^1^Key Laboratory of Animal Genetics, Breeding and Reproduction of Shaanxi Province, College of Animal Science and Technology, Northwest A&F University, Yangling, China; ^2^State Key Laboratory of Reproductive Regulation and Breeding of Grassland Livestock, College of Life Sciences, Inner Mongolia University, Hohhot, China; ^3^Key Laboratory of Animal Reproduction and Germplasm Enhancement in Universities of Shandong, College of Life Sciences, Qingdao Agricultural University, Qingdao, China

**Keywords:** Jining Gray goat, litter size, genomic sequence, signatures of selection, candidate genes

## Abstract

Litter size (LS), an important economic trait in livestock, is so complicate that involves many aspects of reproduction, the underlying mechanism of which particularly in goat has always been scanty. To uncover the genetic basis of LS, the genomic sequence of Jining Gray goat groups (one famous breed for high prolificacy in China) with LS 1, 2, and 3 for firstborn was analyzed, obtaining 563.67 Gb sequence data and a total of 31,864,651 high-quality single nucleotide polymorphisms loci were identified. Particularly, the increased heterozygosity in higher LS groups, and large continuous homozygous segments associated with lower LS group had been uncovered. Through an integrated analysis of three popular methods for detecting selective sweeps (Fst, nucleotide diversity, and Tajima’s *D* statistic), 111 selected regions and 42 genes associated with LS were scanned genome wide. The candidate genes with highest selective signatures included *KIT*, *KCNH7*, and *KMT2E* in LS2 and *PAK1*, *PRKAA1*, and *SMAD9* in LS3 group, respectively. Meanwhile, functional terms of programmed cell death involved in cell development and regulation of insulin receptor signaling pathway were mostly enriched with 42 candidate genes, which also included reproduction related terms of steroid metabolic process and cellular response to hormone stimulus. In conclusion, our study identified novel candidate genes involving in regulation of LS in goat, which expand our understanding of genetic fundament of reproductive ability, and the novel insights regarding to LS would be potentially applied to improve reproductive performance.

## Introduction

Goat (*Capra hircus*), one of the most important livestock animals, has been domesticated from wild goat near Fertile Crescent in Middle East around 10,000 years ago (Naderi et al., 2008; Daly et al., 2018). Accompanied by the migration activity of human, the goat exhibited the strong adaptability to diverse environments, and gradually spread all over the world (Benjelloun et al., 2015). To meet human demand, many functional traits, such as dairy, meat, cashmere yield, and reproductive performance, all underwent a long period of natural selection and artificial intervention, ultimately, some specialized goat breeds with high performance of production were generated, which provided abundant genetic resources for modern domesticated goat.

Litter size (LS, numbers of lambing per ewe) is such a complex trait, which involves multiple biological process, including hormone secretion, follicular growth, ovulation, fertilization, embryo implantation, placenta and fetal development, and so on (Feng et al., 2015; Xu et al., 2018). Among which, the ovulation rate and LS are most important (Fogarty, 2009). Previously, more attentions have been attracted in reproductive trait study of sheep, four major prolificacy (that is LS) genes have been identified, that are bone morphogenetic protein receptor 1B (*BMPR1B*, *FecB*), bone morphogenetic protein 15 (*BMP15*, *FecX*), growth differentiation factor 9 (*GDF9*, *FecG*), and beta-1,4-*N*-acetyl-galactosaminyl transferase 2 (*B4GALNT2*, *Fec*^L^) (Polley et al., 2010; Drouilhet et al., 2013; Våge et al., 2013; Abdoli et al., 2016). In particular, it has been proved an extra ovulation increased per estrus for one copy of *BMP15* (0.4) and *BMPR-1B* (1.5) mutation (Davis, 2005; Abdoli et al., 2016; Talebi et al., 2018). Actually, the mutations of *GDF9* and *BMP15* genes had been verified to be strongly associated with LS trait in sheep (Demars et al., 2013; Våge et al., 2013). Recently, one research of genome-wide association studies (GWAS) in sheep had focused on LS trait, the high- and low-prolificacy sheep breeds were applied to explore the significant variants associated with LS, it revealed amount of candidate single nucleotide polymorphisms (SNPs), including *BMPR1B*, *FBN1*, and *MMP2* in Wadi sheep, *SMAD1* and *CTNNB1* in Hu sheep, *NCOA1* in Icelandic sheep, *FLT1*, *NF1*, *PTGS2*, and *PLCB3* in Finn sheep, *ESR2* in Romanov sheep, and *ESR1*, *ETS1*, *FLI1*, *SPP1*, and *MMP15* in Texel sheep (Xu et al., 2018).

Currently, the genetic research on the characteristics of goat is lagging, and the limited information of LS trait in goat mostly derived from reports of sheep, that major in *BMPR1B*, *BMP15*, and *GDF9* (An et al., 2015a, b; Ahlawat et al., 2016). Besides, there were few association analysis of single gene, for example, the SNPs of POU class 1 homeobox 1 (*POU1F1*) and *KISS1* genes, insertion/deletion (indel) variants of lysine demethylase 6A (*KDM6A*) and β-Type platelet-derived growth factor receptor (*PDGFRB*), and transcript variants of catenin beta 1 (*CTNNB1*) had been reported to be associated with goat LS trait (An et al., 2013; Cui et al., 2018; Zhang X. et al., 2018; Yang et al., 2019; Zhu et al., 2019). Meanwhile, the genome-wide research on goat is also very lacking, only fewer reports showed some novel mutation sites affecting goat reproduction by whole-genome sequence (Guan et al., 2016; Lai et al., 2016). According to Guan et al. (2016), the reproduction-related genes regard to neurohypophysial hormone activity, including *PAIP2B*, *CCDC64*, and *EPB41L5*, had been identified. Also, Lai et al. (2016) revealed a series of candidate genes for fecundity (number of lambs) in Laoshan dairy goats, such as synonymous mutation: *MAP3K12*, *DNMT3B*, and *SMAD2* and non-synonymous mutation: *SETDB2* and *CDH26*. Moreover, *PSEN2* gene, that locates at surrounding transcription start sites, was suggested to be related to goat fecundity, *PRP1* and *PRP6* genes were discovered to be linked to reproductive hormone with copy number variation (CNV) analysis (Zhang R.-Q. et al., 2018; Zhang et al., 2019).

Jining Gray goat, living in Shandong province of China, is one of the most outstanding local breeds in the world, because of its high fertility performance and it has shorter sexual maturity than other breeds (Miao et al., 2016; Su et al., 2018), thus the breed is the optimal animal model for detection of selection signatures associated with reproductive ability in goat. Several reports have investigated the reproductive performance focused on Jining Gray goat from different aspects (Chu et al., 2011; Shi et al., 2015; Miao et al., 2016; Su et al., 2018), but the variant research in genome-wide has not been reported. Actually, as one kind of low heritability traits in livestock, LS has been hard to improve with traditional selection. Presently, the technologies of genomic selection (GS) in livestock have become the most promising power to improve the traits with low heritability (Cm Dekkers, 2012; Jonas and Koning, 2015). Despite of under development, the implementation of GS in livestock has been effective for increasing the accuracy of estimated breeding values (EBVs), by using the genome-wide SNP in breeding programs, it shortens generation intervals and is more economical (VanRaden et al., 2009; Pryce and Daetwyler, 2012). Regard to this, the effective genetic variants associated with breeds advantage genotypes have to be uncovered, especially in goat.

To elucidate the genetic fundament of LS in goat, 40 Jining Gray goats, with LS of 1 (20 individuals), 2 (14 individuals), and 3 (6 individuals) for firstborn, were performed with the whole-genome sequence at ∼10× coverage in this study. In the result, amounts of whole-genome variants, including SNPs and indel, had been discovered. Meanwhile, the selective sweeps of different LS groups were contributed to screen the regions with strong signatures of selection, and the most candidate genes had been identified within Jining Gray goat population, that provided abundant candidate loci for reproduction improvement in domestic goat.

## Materials and Methods

### Ethics Statement

All goats in this study were keep feeding in Caoxian Zhengdao Jining Gray goat’ farm in Heze, Shandong, China. The experiment procedures were approved by the Ethics Committee of Northwest A&F University.

### Animals

The selected goats were 2∼3 years old female individuals, and the natural mating was arranged with normal reproductive male goats. And the LS information was derived from record of Caoxian Zhengdao Jining Gray goat farm. Total 40 Jining Gray goats were divided into three groups with LS of 1 (20 individuals), 2 (14 individuals), and 3 (6 individuals) for firstborn.

### Samples Collection, DNA Extraction, and Whole-Genome Sequence

Ear tissues of selected goats were collected in farm, transferred in sterile centrifuge tube, and stored in library at −80°C. For sequence, the samples were transferred with dry ice to the Beijing Novogene Bioinformatics Technology Co., Ltd. The DNA sample of each individual was extracted and measured following standard experimental procedures to guarantee their quantity. Subsequently, the DNA samples were interrupted randomly to produce fragments of 350 bp, and the library was constructed with a series of steps of end repair, ployA tailed, purification, PCR amplification, and so on. The whole-genomic sequence with paired-end 150 bp strategy was performed by the Illumina NovaSeq 6000 platform (Illumina Inc., San Diego, CA, United States).

### Genomic Data Processing and Variants Calling

With the sequence data from company, the Trimmomatic software (version 0.38) (Bolger et al., 2014) was applied to remove adaptors and low-quality reads, and FastQC reports (version 0.11.6) (Andrews, 2010) were generated to check data quality (for statistics information, see Supplementary Table S2). Through BWA software (version 0.7.17) (Li and Durbin, 2009), the clean data were aligned to goat reference genome^1^ (ARS1) (Bickhart et al., 2017), and the bam and sorted bam files were generated with samtools (version 1.9) (Li et al., 2009). Then, the Genome Analysis Toolkit (GATK, version 4.1.3.0) (McKenna et al., 2010) pipeline was executed with diverse modules for variants calling: briefly, it was prepared with duplicates fix of sorted bam files, then “HaplotypeCaller” module produced the raw variants of gVCF files, and “CombineGVCFs” was carried to merge all raw results as one gVCF file, then “GenotypeGVCFs” module had been operated to consolidate the calling variants to generate VCF file (including SNPs and indels). Finally, the preliminary SNPs and indels were produced with the VCF file by “SelectVariants” mode.

### Quality Control and Annotation for Detected SNPs

To guarantee the quality, the raw SNPs were filtered by a series of thresholds with “VariantFiltration” mode of GATK. That is: “QD (Quality score by depth) >2.0 & MQ (Mapping quality score) >40.0 & FS (Phred-scaled *p*-value using Fisher’s exact test) <60.0 & ReadPosRankSum (Rank Sum Test for relative positioning of reference and alternative alleles within reads) > −8.0 & MQRankSum (Rank sum test for mapping quality of reference and alternative within reads) > −12.5,” which had been widely used to generate the high-quality SNPs for subsequent analysis. To identify the variants function, ANNOVAR software was utilized to annotate the filtered SNPs (version 2018-04-16) (Wang et al., 2010). The SNPs were displayed with circos plot with shinyCircos^2^ (Yu Y. et al., 2017) and density plot by CMplot package^3^ in R environment (v 3.6.0).

### Population Genetics and LD Analysis

For principal component analysis (PCA), the software of plink (v1.90b4) (Purcell et al., 2007) was performed to prepare the input file (.bed, .bim, .fam) and conduct simple filtering (–geno 0.05), with autosomal SNPs of 95% genotyping rate, gcta64 (v 1.26.0) (Yang et al., 2011) was applied to calculate genetic relationship matrix (–make-grm) and principal component (–pca). And plot was generated with python in local. The neighbor-joining (NJ) phylogenetic tree was constructed by MEGA (v 10.0.5) (Saitou and Nei, 1987; Kumar et al., 2018) software based on the genetic distance-matrix calculating by plink and plotted with iTOL (v 4.4.2) (Letunic and Bork, 2019). Also, the linkage disequilibrium (LD) decay of *r*^2^ (a measure of LD, that is squared coefficient of correlation (r) between loci) was carried out with PopLDdecay with general parameter (Zhang C. et al., 2018).

### Runs of Homozygosity

The runs of homozygosity (ROH) value was estimated with autosomal SNPs of each individual by plink (v1.90b4). In our research, six criteria were set to define a ROH: a window size of 50 SNPs for scanning; the minimum length of ROH was 200 kb; the required minimum density was 50 kb/SNP; the maximum gap between consecutive homozygous SNPs was 500 kb length; a scanning ROH can contain at most one heterozygous and five missing genotypes; threshold of all scanning windows containing SNP was 0.05. As for assessment, ROH were divided into five categories based on length: 0–0.5 Mb, 0.5–1 Mb, 1–2 Mb, 2–4 Mb, and >4 Mb. All the parameter setting and classification for assessment had referenced other published reports (Peripolli et al., 2017; Onzima et al., 2018; Xu et al., 2019).

### Detection of Selective Signatures

In preliminary, the values of pairwise Fst, heterozygosity, allele frequency, nucleotide diversity and Tajima’s *D* were calculated by vcftools with parameter of “*--*weir-fst-pop,” “*--*het,” “*--*freq,” “*--*window-pi,” and “*--*TajimaD” (v 0.1.16) (Danecek et al., 2011). For heterozygosity rate, it was calculated by the formula of (N_SITES-O(HOM))/N_SITES, O(HOM) represents the observed number of homozygous sites of each individual, N_SITES is the total number sites of each individual. With comparisons between diverse LS groups, the selective sweeps analysis was performed according the genetic differentiation (Fst), nucleotide diversity, and Tajima’s *D* values, which were computed with 100 kb window size and 50 kb step size. And the per window Fst was standardized to Z-transformed Fst [Z(Fst)] based on the formula of “per window Z(Fst) = (per window Fst − mean Fst of all windows)/standard deviation of all windows Fst.” The threshold of Z(Fst) value = 5 was applied to detect the selective regions. Moreover, the highly selected regions were supported by Tajima’s *D* values of different LS groups and pairwise nucleotide diversity (π). Manhattan plots of Z(Fst) value for comparisons between groups were made with the R package: qqman (Turner, 2014).

### Functional Annotation for Candidate Genes

To uncover the biological functions of candidate genes, we performed the Gene Ontology (GO) and Kyoto Encyclopedia of Genes and Genomes (KEGG) enrichment analysis with Metascape (Zhou et al., 2019), a web-based portal for functional enrichment.^4^ Due to the inadequate database of enrichment analysis in goat, the goat gene symbol IDs were converted into mouse homologous gene symbol IDs through Ensembl website.

## Results

### Sequence and Variants Detection of Jining Gray Goat

**FIGURE 1:**
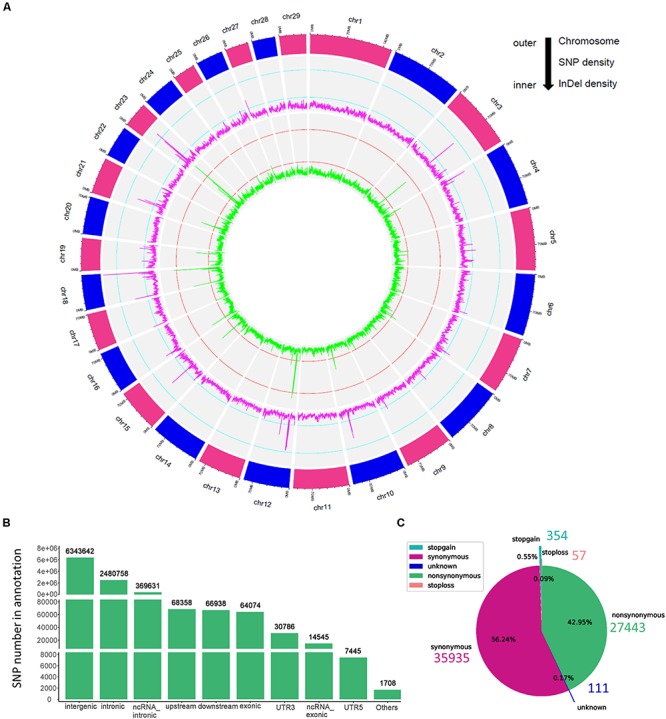
The genomic variants identified and annotated in Jining Gray goat. **(A)** Circos plot of variants density after filtration. The density of variants was calculated in each 100 kb step size. From outer to inner, the outermost circos depicts each chromosome, the middle displayed the SNP density per window (magenta), and the internal circos meant the indel density per window (green). The cyan and red lines in secondary and third cycle was indicated the 0.25 and 0.75 stack height, respectively. **(B)** Genomic annotation of SNPs according to ANNOVAR. “Others” contained a few other categories of “upstream; downstream,” “splicing,” “ncRNA_splicing,” “exonic; splicing,” “UTR5; UTR3,” and “ncRNA_exonic; splicing.” **(C)** The pie plot of annotated SNPs at exonic regions.

In the present study, we sequenced 40 Jining Gray goats, which were divided into three groups with LS values of 1, 2, and 3 for firstborn. The total 563.67 Gb raw data was generated with average sequence depth >8 of each individual sample (Supplementary Tables S1, S2), and mean depth of LS groups mainly ranged from 9 to 11 (Supplementary Figure S1A). With high mapping rate (over 99%) to reference genome, we called 32,072,110 SNPs, after filtered, we got 31,864,651 SNPs with high quality (Table 1). Among which, the substitution mutations with largest number were C > T and G > A, secondly, A > G and T > C followed (Table 1). Moreover, the densities of SNPs and indels spreading on chromosomes were displayed with circos plot (Figure 1A), which indicated the maximum density of SNPs and indels occurred on chromosomes 18 and 23, respectively.

**TABLE 1:** SNP statistical information of Jining Gray goat.

**Classification**	**SNP count**	**Category**		**SNP count**	
After calling	32,072,110	Intergenic		6,343,642	
After filtered	31,864,651	Intronic		2,480,758	
**Substitution types**	**SNP count**	ncRNA_intronic		369,631	
A > C	1,301,360	Upstream		68,358	
A > G	4,653,435	Downstream		66,938	
A > T	1,190,733	Exonic		64,074	
C > A	1,489,351	UTR3		30,786	
C > G	1,288,407	ncRNA_exonic		14,545	
C > T	6,112,390	UTR5		7,445	
G > A	6,124,624	Upstream; downstream		1,271	
G > C	1,298,941	Splicing		287	
G > T	1,483,527	ncRNA_splicing		72	
T > A	1,197,834	Exonic; splicing		42	
T > C	4,644,892	UTR5; UTR3		29	
T > G	1,294,533	ncRNA_exonic; splicing		7	

**Exonic variant**	**Synonymous**	**Non-synonymous**	**Stopgain**	**Stoploss**	**Unknown**

SNP count	35,935	27,443	354	57	111

Moreover, the high-quality SNPs in autosome reached 28,926,281 Supplementary Figure S1B), and the SNPs number within 1 Mb window size was mainly around 8,000 and high-density SNPs (more than 16,000 in each window) largely distributed at the ends of chromosomes (Supplementary Figure S1C). With annotation, we got 9,447,885 SNPs variant results (29.65% of total) and 8,878,492 at autosome (Supplementary Figure S1B), which meant about two-thirds were novel SNPs in goat. These SNPs were partitioned into intergenic (6,343,642; 67.14%), intronic (2,480,758; 26.26%), exonic (64,074; 0.68%) and other gene regulatory regions (Figure 1B and Table 1). For SNPs in exonic regions, it showed the overwhelming majorities were synonymous and non-synonymous, and others including stopgain, stoploss, and unknown were <1% (Figure 1C and Table 1).

### Divergent Genetic Differentiation of Jining Gray Goat LS Groups

**FIGURE 2:**
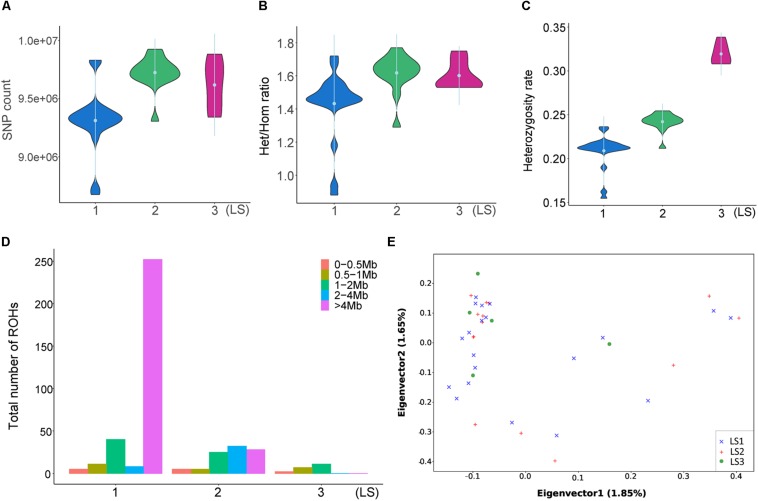
Divergent genetic differentiation of Jining Gray goat groups associated with LS. **(A)** SNPs number and Het/Hom ratio **(B)** of LS1, LS2, and LS3 groups. **(C)** Heterozygosity analysis of LS1, LS2, and LS3 groups. **(D)** Spectrum of ROH length of diverse LS groups. **(E)** Principal component analysis (PCA) of LS1, LS2, and LS3 groups.

To disclose the genetic differences between groups with different LS values, the SNPs of diverse LS groups were investigated. As showed in Figure 2A, there were more SNPs in LS2 than LS1 group, and LS3 group was medium. Similarly, the heterozygous/homozygous (het/hom) ratios in LS2 and LS3 were a little bit higher than that of LS1 group (Figure 2B), as well as the heterozygosity rates of different groups (Figure 2C). ROH analysis further revealed large ROHs of >4 Mb were most associated with lower LS group (Figure 2D), that supported the increase of heterozygosity rates in high LS groups. Moreover, we performed PCA based on SNPs in autosomes (Figure 2E), indicating these samples had no obvious structure regarding the LS groups, which was consistent with the NJ tree result (Supplementary Figure S2A). Further, the LD analysis with LD decay of *r*^2^ showed there was no apparent difference among the three LS groups (Supplementary Figure S2B), which was likely because these individuals come from the same large population.

### Signatures of Selection With Sweeps Analysis

**FIGURE 3:**
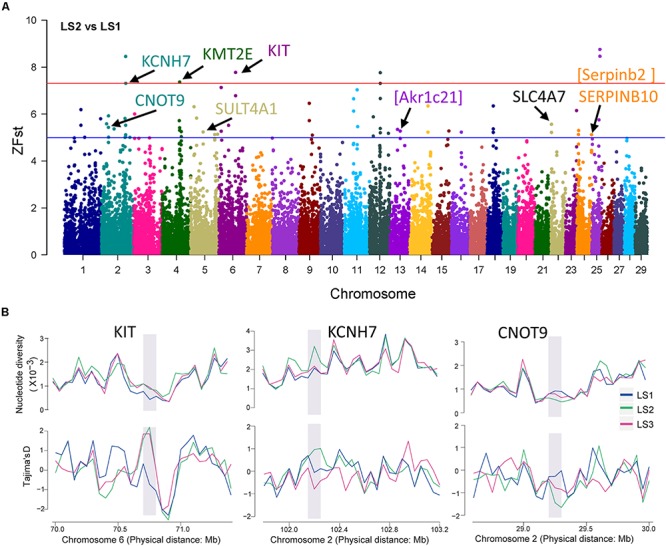
Selective sweep of Jining Gray goat groups with LS2 vs LS1. **(A)** Mahattan Plot of Z(Fst) values of LS2 vs LS1 group. The blue line is “suggestiveline,” –log10(1e – 05), red line is “genomewideline,” –log10(5e – 08). **(B)** Distribution of Tajima’s *D* values and π in LS1, LS2, and LS3 groups at selective regions. The selective regions were marked with gray shadow.

To detect signatures of selection associated with LS, the genetic differentiation was estimated with the fixation index (Fst) in groups of Jining Gray goat (Supplementary Table S3). Firstly, genome-wide SNPs in autosome were utilized to calculate Fst value of LS2 vs LS1 groups, which was performed with 100 kb window size and 50 kb step size. As previous reports, the regions with high Z-transformed Fst values [Z(Fst) > 5] were concentrated (Axelsson et al., 2013; Gou et al., 2014). Here, total 67 windows [Z(Fst) > 5] had been strongly selected (Figure 3A), where *KCNH7*, *KMT2E*, and *KIT* genes located, other genes, consist of *CNOT9*, *SULT4A1*, and *SERPINB10*, underwent subordinate selection (Table 2 and Supplementary Table S4). Especially, there were some novel genes in goat, part of which were generated with the homologous in mouse, i.e., *Akr1c21* and *Serpinb2* (Figure 3A). Genes, *KIT*, *KCNH7*, and *CNOT9*, were verified with Tajima’s *D* values and π (pairwise LS2 vs LS1) (Figure 3B). Allele frequencies for these loci are shown in Supplementary Figure S2C. Moreover, the non-coding region of *KIT* and *KCNH7* also possessed many mutations (Supplementary Figures S3A,B). Among, *KIT* gene was most likely the candidate gene in LS2 group of Jining Gray goat.

**TABLE 2:** Gene located in selective regions of LS2 vs LS1 and LS3 vs LS1 with Z(Fst) > 5.

**Chromosome**	**Z(Fst)**	**Gene name**	**Description (if have)**
**Top selective regions of LS2 vs LS1**			
25	8.461866	Novel	2-Acylglycerol *O*-acyltransferase 3-like
25	8.461866	Novel	
2	8.454405	KCNH7	Potassium voltage-gated channel subfamily H member 7
6	7.773884	KIT	KIT proto-oncogene, receptor tyrosine kinase
4	7.363256	KMT2E	Lysine methyltransferase 2E
1	6.18886	Novel	Mucin 4, cell surface associated
3	6.005006	[Lrrfip1]	LRR binding FLII interacting protein 1
22	5.564981	SLC4A7	Solute carrier family 4 member 7
1	5.522897	TNK2	Tyrosine kinase non-receptor 2
11	5.463277	[Ebag9]	
2	5.432469	CNOT9	CCR4-NOT transcription complex subunit 9
4	5.310074	[Rpl7a]	60S ribosomal protein L7a pseudogene
24	5.299603	RTTN	Rotatin
13	5.275389	[Akr1c21]	Dihydrodiol dehydrogenase 3
5	5.139774	SULT4A1	Sulfotransferase family 4A member 1
5	5.139774	PNPLA5	Patatin like phospholipase domain containing 5
5	5.12483	Novel	Antigen WC1.1-like
5	5.12483	Novel	
24	5.121225	[Serpinb2]	Plasminogen activator inhibitor 2
24	5.121225	SERPINB10	Serpin B10
9	5.09999	ENPP3	Ectonucleotide pyrophosphatase/phosphodiesterase 3
9	5.09999	ENPP1	Ectonucleotide pyrophosphatase/phosphodiesterase 1
2	5.03017	IFIH1	Interferon induced with helicase C domain 1
2	5.014631	[Cxcr2]	
2	5.014631	[Rufy4]	
2	5.006772	[Baz2b]	Bromodomain adjacent to zinc finger domain 2B
**Top selective regions of LS3 vs LS1**			
20	8.751742	CARD6	Caspase recruitment domain family member 6
20	8.751742	PRKAA1	Protein kinase AMP-activated catalytic subunit alpha 1
9	7.775685	TTK	TTK protein kinase
29	7.294919	PAK1	p21 (RAC1) activated kinase 1
21	6.301782	PSMA6	Proteasome subunit alpha 6
29	6.080819	ZDHHC13	Zinc finger DHHC-type containing 13
29	5.80846	[Gdpd4]	Glycerophosphodiester phosphodiesterase domain containing 4
2	5.652142	PARD3B	Par-3 family cell polarity regulator beta
19	5.633573	TANC2	Tetratricopeptide repeat, ankyrin repeat, and coiled-coil containing 2
28	5.55818	PSAP	Prosaposin
28	5.55818	CDH23	Cadherin related 23
9	5.451982	LCA5	Lebercilin LCA5
15	5.398031	SORL1	Sortilin related receptor 1
9	5.396861	ELOVL4	ELOVL fatty acid elongase 4
12	5.302314	SMAD9	SMAD family member 9
12	5.302314	ALG5	ALG5 dolichyl-phosphate beta-glucosyltransferase
14	5.146363	PRKDC	Protein kinase, DNA-activated, catalytic subunit
14	5.146363	[H3f3b]	Histone H3.3C
29	5.125145	PRMT3	Protein arginine methyltransferase 3
19	5.124549	[Pctp]	Phosphatidylcholine transfer protein
17	5.117664	ATXN2	Ataxin 2

**FIGURE 4:**
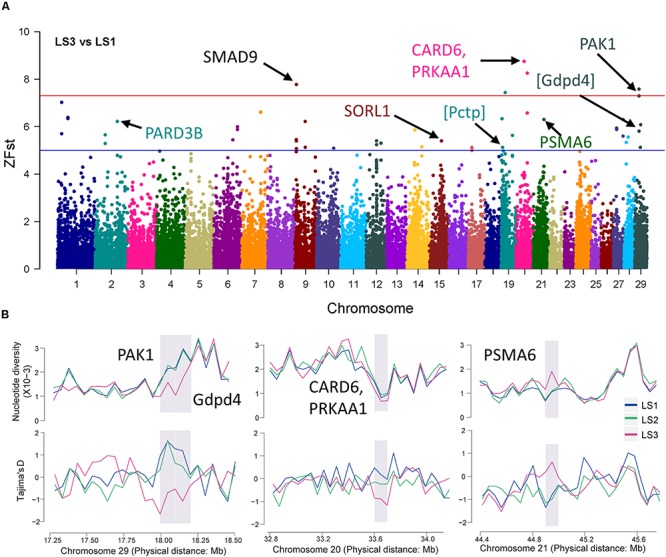
Selective sweep of Jining Gray goat groups with LS3 vs LS1. **(A)** Mahattan Plot of Z(Fst) values of LS3 vs LS1 groups. The blue line is “suggestiveline,” –log10(1e – 05), red line is “genomewideline,” –log10(5e – 08). **(B)** Distribution of Tajima’s *D* values and π in LS1, LS2, and LS3 groups at selective regions. The selective regions were marked with gray shadow.

Further, the selective sweep analysis also was executed in LS3 vs LS1 groups (Figure 4A and Table 2). Here, we obtained 44 regions with highest Z(Fst) value above the genome-wide line (Supplementary Table S3), which located at chromosome 9, 20, and 29, and the selection genes of *SMAD9*, *CARD6* and *PRKAA1*, and *PAK1* were annotated, respectively. Also, the genes at a sub-optimal level included *PARD3B*, *SORL1*, and *PSMA6* (Table 2 and Supplementary Table S4), especially. Several novel LS genes in goat were mapped in mouse orthologs genes, such as *Pctp* and *Gdpd4*. Moreover, these genes in selective regions of LS3 group, *PAK1* and *Gdpd4*, *CARD6* and *PRKAA1*, and *PSMA6*, were partly confirmed by Tajima’s *D* and π values compare to LS1 group (Figure 4B), that also had been reflected by the changes of allele frequency at mutation loci (Supplementary Figure S2D). In addition, we discovered that *PAK1* gene had several mutations in non-coding region (Supplementary Figure S3C). These results indicted *PAK1* was mostly candidate gene in LS3 group of Jining Gray goat.

### Functional Enrichment of Candidate Genes

**FIGURE 5:**
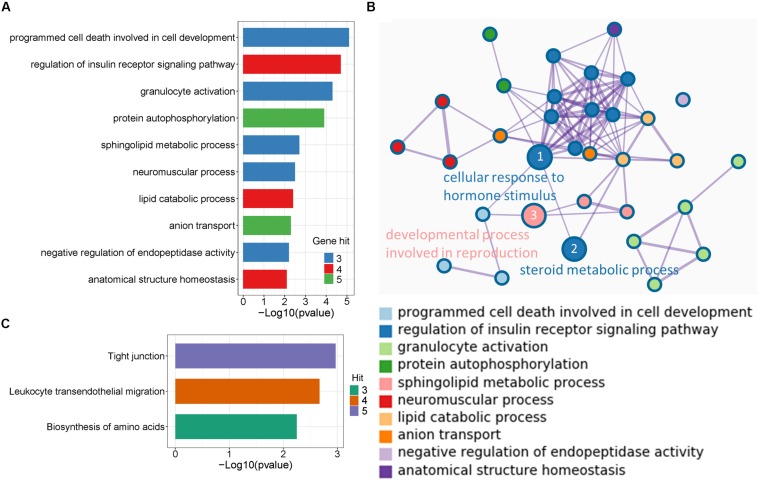
Enrichment analysis of candidate genes. **(A)** The top non-redundant go enrichment clusters of genes in selected region both of LS2 vs LS1 and LS3 vs LS1 groups. Per cluster use a discrete color scale to represent statistical significance. **(B)** Metascape enrichment network showing the intra-cluster and inter-cluster similarities of enriched go terms. Node size is reflected by gene hits in the go term. **(C)** Enriched KEGG pathway of candidate genes.

To disclose the function of candidate genes associated with LS trait, the GO enrichment analysis of annotated genes in regions with Z(Fst) > 5 both LS2 vs LS1 and LS3 vs LS1 groups was performed (Table 3). The most top of non-redundant enrichment GO terms were “programmed cell death involved in cell development” and “regulation of insulin receptor signaling pathway” (Figure 5A). Of note, enrichment map of all GO lists showed “steroid metabolic process,” “cellular response to hormone stimulus,” and “developmental process involved in reproduction” were also enriched with more gene hits (marked with numbers) (Figure 5B and Supplementary Table S5), all these processes were likely evidenced the genotype of the high LS of firstborn in Jining Gray goat. Meanwhile, the enriched pathway of “tight junction” might also serve as other important roles in high LS (Figure 5C and Table 4).

**TABLE 3:** Top non-redundant enriched GO terms of candidate genes.

**GO**	**Description**	**LogP**	**Gene in GO**	**Hits**
GO:0010623	Programmed cell death involved in cell development	−5.1	3	Kit| Prkdc| Slc4a7
GO:0046626	Regulation of insulin receptor signaling pathway	−4.7	4	Pak1| Enpp1| Sorl1| Prkaa1
GO:0036230	Granulocyte activation	−4.3	3	Cxcr2| Kmt2e| Enpp3
GO:0046777	Protein autophosphorylation	−3.9	5	Kit| Pak1| Enpp1| Ttk| Tnk2
GO:0006665	Sphingolipid metabolic process	−2.7	3	Kit| Psap| Elovl4
GO:0050905	Neuromuscular process	−2.5	3	Psap| Atxn2| Cdh23
GO:0016042	Lipid catabolic process	−2.4	4	Psap| Sorl1| Pnpla5| Prkaa1
GO:0006820	Anion transport	−2.3	5	Pak1| Pctp| Enpp1| Psap| Slc4a7
GO:0010951	Negative regulation of endopeptidase activity	−2.2	3	Serpinb2| Sorl1| Serpinb10
GO:0060249	Anatomical structure homeostasis	−2.1	4	Prkdc| Cdh23| Lca5| Prkaa1

**TABLE 4:** Enriched KEGG pathways of candidate genes.

**Category**	**Description**	**LogP**	**Gene in pathway**	**Hits**
Pathway	Biosynthesis of amino acids	−2.25	3	Aldoc| Gpt| Tat
Pathway	Leukocyte transendothelial migration	−2.67	4	Actb| Cldn8| Rapgef4| Cldn17
Pathway	Tight junction	−2.97	5	Actb| Dlg2| Cldn8| Pard3| Cldn17

## Discussion

In this study, we provided the comprehensive evidence of genetic background of high fecundity in goat. Briefly, the whole-genomic sequence was performed with groups of Jining Gray goat (one famous Chinese local breed with mean LS 2.94) with LS 1, 2, and 3 for first-born and we got a plenty of genome-wide variants. Most of genomic SNPs in Jining Gray goat were identified for the first time in our study. Importantly, signatures of selection among LS groups were uncovered with selective sweeps of Fst, π, and Tajima’s D. We identified 42 candidate genes associated with LS trait, which was likely contribute for GS in goat to improve the reproductive performance.

As previously reported, follicular maturation and ovulation rate are the most important factors affecting LS, increasing the possibility of higher fertilized rate and bigger litters (Fogarty, 2009). It has been suggested that the members of transforming growth factor-β (TGF-β) superfamily, including *BMPR-1B*, *BMP15*, and *GDF9*, affected the ovulation and LS (Demars et al., 2013). Also, numerous studies had explored the polymorphisms of these genes in sheep and goat breeds (Ahlawat et al., 2016; Talebi et al., 2018). Here, we identified large amounts of SNPs in Jining Gray goat, and more than half were novel sites, all the abundant variants extended genetic resource in goat for fecundity traits associated research. Of note, there were no distinct grouped patterns in PCA and NJ tree results, as well as LD decay analysis, which might cause by the similar genetic background within population. Recently, ROH in animals present adamant evidence of individual relatedness, inbreeding, and also selection pressure (Peripolli et al., 2017; Ceballos et al., 2018). Our results showed that large continuous homozygous segments were mostly associated with lower LS group, which displayed consistent with increase of heterozygosity rate in higher LS groups, these results indicated the difference of heterozygosity in diverse LS groups was likely related to fitness and inbreeding.

For genomic research, it has been established that GWAS is the most powerful tools for detecting the genetics basis of complex traits (McCarthy et al., 2008; Stranger et al., 2011). Accordingly, an adequate statistical power of GWAS require a much larger sample size, and the proper case-control design is relative necessary (Hong and Park, 2012). However, these very strict requirements of the large sample size and detailed genealogy information prevent us to carry out the similar study in goat, regard to their availability. At the same time, the threshold character of LS trait had been reported as previously (Konstantinov et al., 1994; Pérez-Enciso et al., 1995; Janssens et al., 2004), and the extreme phenotypic study design is also much more powerful to detect the variants associated trait selection (Peloso et al., 2016), thus, the sweeps analysis of pairwise value between LS groups were conducted.

In our study, a total of 42 candidate genes associated with LS trait were identified by selective sweeps of three groups within Jining Gray goat. In LS2 group, the genes of *KCNH7*, *KMT2E*, and *KIT* possessed most strong selective signatures. *KIT*, is known to be involved in oocyte growth and follicular development in mammals, deficient of *KIT* would lead amounts of oocytes lost (Moniruzzaman et al., 2007), and evidence showed mutations of *KIT* or *KITLG* blocked the interaction of oocyte and granulosa cell, and caused female infertile (Horie et al., 1991; Driancourt et al., 2000; Thomas et al., 2008). Moreover, *KITLG* has been previously identified to be associated with LS trait in goat (An et al., 2015a, b), such evidence suggested *KIT* may be the most promising gene for large LS. Another, *KCNH7* also had been found to be significantly associated with animal reproductive traits (Fan et al., 2017). Besides, *KMT2E*, termed as *MLL5*, had been demonstrated to be required for normal spermatogenesis, its knockout mouse showed a post meiotic phenotype and infertile (Heuser et al., 2009; Yap et al., 2011).

Besides, in LS3 group, the genes of *SMAD9*, *CARD6*, *PRKAA1*, and *PAK1* possessed most strong selective signatures. Among which, *SMAD9* (also known as *SMAD8*), a member of the *SMAD* family, involved signal transduction of TGF-β superfamily and inhibited BMP activity (Chang et al., 2002; Tsukamoto et al., 2014), it had already been suggested to control follicular selection via balancing luteinizing hormone receptor (LHR) transcription (Yu D. et al., 2017). Similarly, *SMAD2* in goat and *SMAD1* in sheep also were detected to be associated with LS trait (Lai et al., 2016; Xu et al., 2018). In addition, *PAK1*, a serine/threonine kinase, had been reported to mediate estradiol-negative feedback actions in the reproductive axis, which seems to play important roles in non-classical estrogen receptor α (ERα) signaling (Zhao et al., 2009). *PRKAA1*, protein kinase AMP-activated catalytic subunit alpha 1, served as an energy sensor that maintains energy homeostasis (Kahn et al., 2005; Krishan et al., 2014). And another gene, *PARD3B* with lower selection intensity here, had been identified with goat productive traits in another report (Guan et al., 2016).

Especially, our results suggested regions detected with Fst were not supported by Tajima’s *D* or π. These contradictory results may be caused by differences in the underlying theory among methods. Firstly, Fst, is developed for population differentiation, it compares the variance of allele frequencies within and between populations, and is suggestive of directional selection (Holsinger and Weir, 2009). Another, Tajima’s *D* is calculated based on frequency spectrum, when selection occurs, a population wide reduction in the genetic diversity follows, and many surrounding sites near the selected variant segregate at low frequencies, then the frequency spectrum shifts back to baseline over thousands of generations (Tajima, 1989). Conversely, population differentiation-based approaches (that is Fst) can detect more types of selection, including classic sweeps, sweeps on standing variants, and negative selection (Vitti et al., 2013). Thus, we believed the result of Fst was potentially acceptable, although they might be not suggested by other values at secondary level of significance. Moreover, the inconsistencies between results of LS2 vs LS1 and LS3 vs LS1 attracted more attentions. Here we may hypothesize the LS trait is not only quantitative, but also a threshold trait, it means different major genes and more minor genes when LS increase. In our result, there were no shared candidate genes between in LS2 and LS3 groups, it seemed to indicate the character of threshold trait of LS, in particular, the allele frequency of *PAK1* steadily increased from LS 1 to 3, which suggested a quantitative trait of LS, although it only showed strong selective signal in LS3 group. However, the underlying mechanism needs further investigation.

Further, the functional enrichment analysis suggested the processes of programmed cell death involved in cell development, cellular response to hormone stimulus, steroid metabolic process and regulation of insulin receptor signaling pathway might play important roles in the regulation of LS trait. Notably, the steroid production is critical for body development and function through steroid hormones, consist of androgen, estradiol, progesterone, glucocorticoid, and aldosterone (Jamnongjit and Hammes, 2006; Ruiz-Cortés, 2012). There were six genes enriched in steroid metabolic process (Supplementary Table S5), *KIT* and *PRKAA1* both with high selective signals that exhibited reproduction or energy-related regulation. In another aspect, programmed cell death plays a fundamental role in animal development, and plays a prominent role in development of fetal ovaries and postnatal ovarian cycle (Pru and Tilly, 2001; Fuchs and Steller, 2011). Moreover, the insulin signal that interacted with steroid metabolic process and cellular response to hormone stimulus was believed to be closely connected with folliculogenesis and ovarian function (Kezele et al., 2002; Dupont and Scaramuzzi, 2016).

Collectively, this research identified numerous novel variants in Jining Gray goat. Some candidate genes showed strongest differentiation signals, such as *KIT*, *PAK1*, *PRKKA1*, *SMAD9*, *KCNH7*, and *KMT2E*. Especially, *KIT* and *PAK1* genes are mostly suggested to be applied to improve the reproductive performance of goats. Meanwhile, the steroid metabolic process and cellular hormone response enriched most genes at selective regions, which would help to explain the difference of LS trait in Jining Gray goat. With regard to application, we believe two steps should be considered: (1) GWAS with large sample size in goat to verify the accuracy of candidate genes; (2) establishment of the core breeding population based on genetic marker genes and reference population expansion. Accordingly, GS is a promising development in livestock breeding programs (Jonas and Koning, 2015), whose implementation of is based on the genome-wide markers of interested trait to conduct genomic EBVs, with daughter assessment, the selected individuals are arranged to reference population. Above of all, our findings will increase the genetic cognition of goat LS trait, contribute to understand the changes of genomic phenotypes under the adaption and artificial breeding process, which would benefit the improvement of reproduction performance in goat.

## Data Availability Statement

The genome sequence data of 40 Jining Gray goats in this research had been submitted in NCBI Sequence Read Archive (SRA) under BioProject No. PRJNA560446.

## Ethics Statement

The animal study was reviewed and approved by the Northwest A&F University. Written informed consent was obtained from the owners for the participation of their animals in this study.

## Author Contributions

J-JW, TZ, Q-MC, R-QZ, LL, and S-FC conducted the experiments. J-JW, TZ, and Q-MC analyzed the data. J-JW, WS, and C-ZL wrote the manuscript. WS and C-ZL designed the manuscript. All authors revised the manuscript and approved the final manuscript.

## Conflict of Interest

The authors declare that the research was conducted in the absence of any commercial or financial relationships that could be construed as a potential conflict of interest.

## Footnotes

1 ftp://ftp.ensembl.org/pub/release-95/fasta/capra_hircus/dna/Capra_hircus.ARS1.dna.toplevel.fa.gz

2 https://github.com/venyao/shinyCircos.git

3 https://github.com/YinLiLin/R-CMplot

4 http://metascape.org/gp/index.html

## Supplementary Material

FIGURE S1 Genomic sequence and variants statistics. **(A)** Mean depth of sequence in LS1, LS2, and LS3 groups. **(B)** Statistics of SNP number in filtration and annotation. **(C)** The number of SNPs within 1 Mb window size at autosome.

FIGURE S2 Structure analysis, LD decay, and allele frequency of different LS groups. **(A)** Circle diagram of NJ tree of all samples. The green panel labeled the LS2 group, and magenta panel labeled LS3 group. **(B)** LD decay plot of LS1, LS2, and LS3 groups. *r*^2^ was calculated with same sample size of different groups (individual = 6). **(C,D)** Allele frequency of mutation loci of candidate genes for LS2 vs LS1 **(C)** and LS3 vs LS1 **(D)**.

FIGURE S3 Structure of protein coding region of most candidate genes from Ensembl website. **(A)** Protein coding regions (left) and genomic mutation count (right) of *KIT* gene. **(B)** Protein coding regions (left) and genomic mutation count (right) of *KCNH7* gene. **(C)** Protein coding regions (left) and genomic mutation count (right) of *PAK1* gene.

TABLE S1 Statistics of reads depth and overage of each sample.

TABLE S2 Number of reads in quality control and mapping statistics.

TABLE S3 The selected regions in LS2 and LS3.

TABLE S4 Mutation loci of candidate genes in both LS2 vs LS1 and LS3 vs LS1.

TABLE S5 All GO lists of candidate genes.
