# Supplementary figures and images for: Genomic Signatures of Selection Associated With Litter Size Trait in Jining Gray Goat
Source: Front Genet. 2020 Mar 26;11:286. doi: 10.3389/fgene.2020.00286 (PMC7113370; doi:10.3389/fgene.2020.00286)

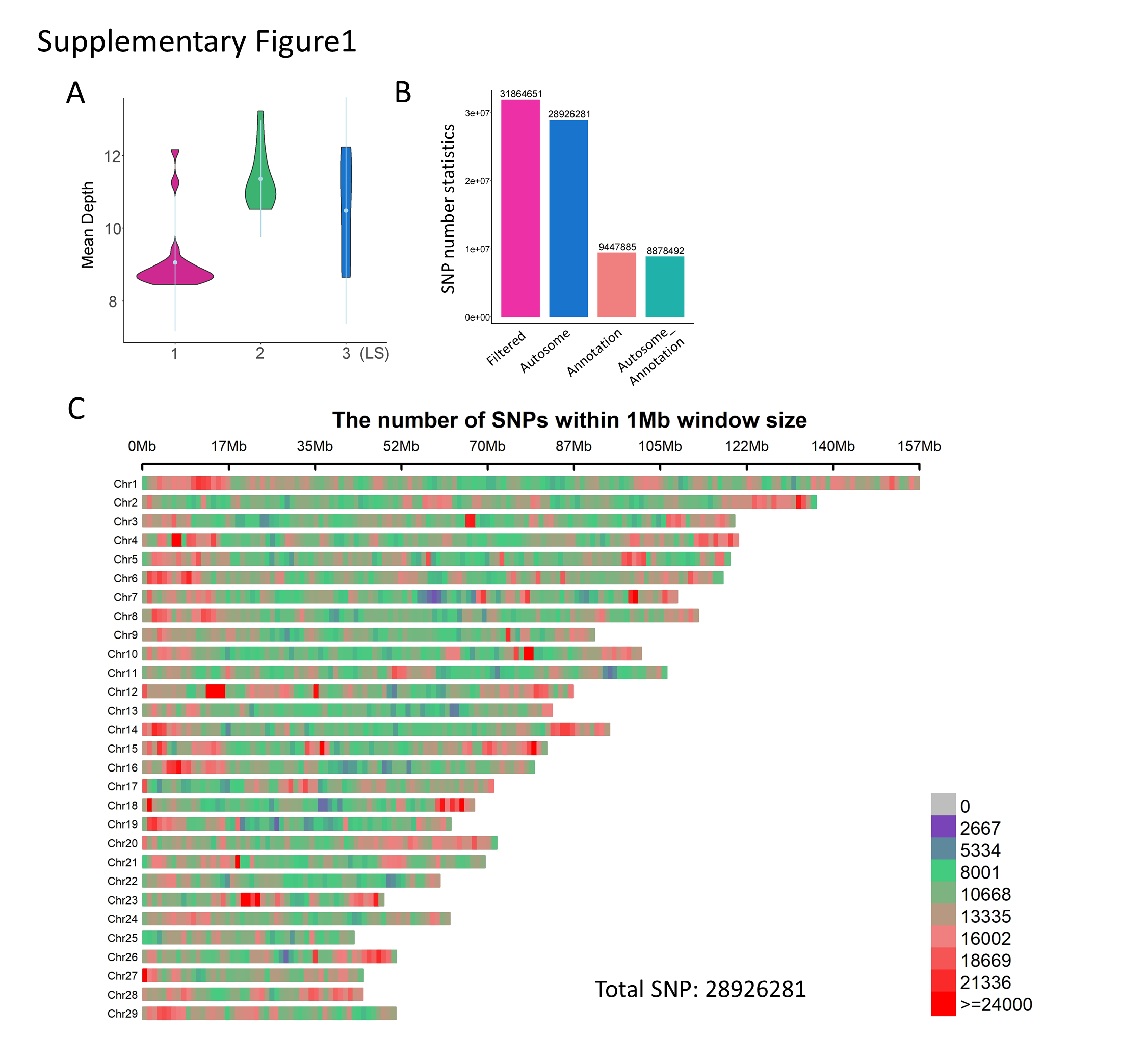

Supplement: FIGURE S1 — Genomic sequence and variants statistics. (A) Mean depth of sequence in LS1, LS2, and LS3 groups. (B) Statistics of SNP number in filtration and annotation. (C) The number of SNPs within 1 Mb window size at autosome. [file Image_1.TIF]

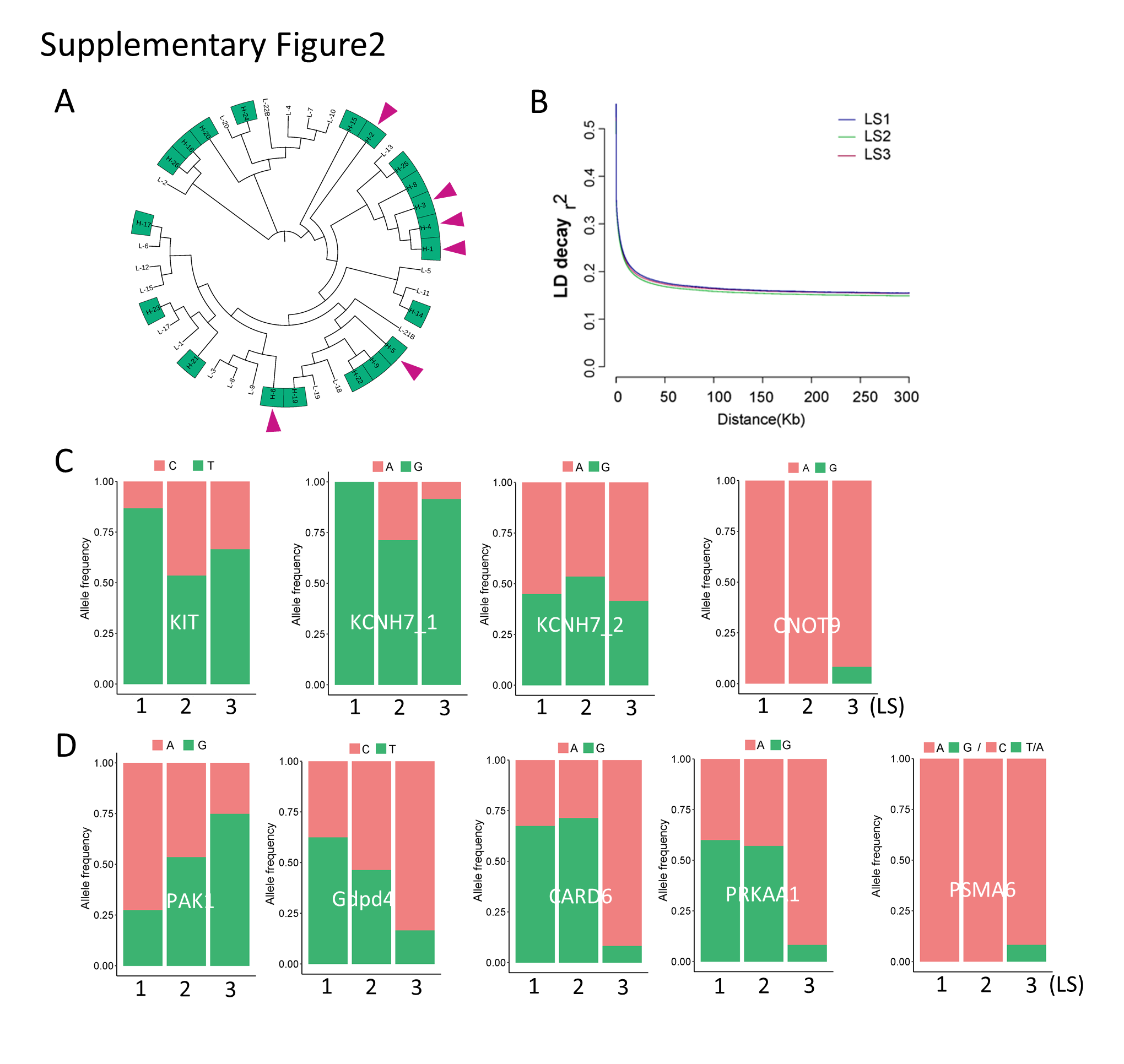

Supplement: FIGURE S2 — Structure analysis, LD decay, and allele frequency of different LS groups. (A) Circle diagram of NJ tree of all samples. The green panel labeled the LS2 group, and magenta panel labeled LS3 group. (B) LD decay plot of LS1, LS2, and LS3 groups. r2 was calculated with same sample size of different groups (individual = 6). (C,D) Allele frequency of mutation loci of candidate genes for LS2 vs LS1 (C) and LS3 vs LS1 (D). [file Image_2.TIF]

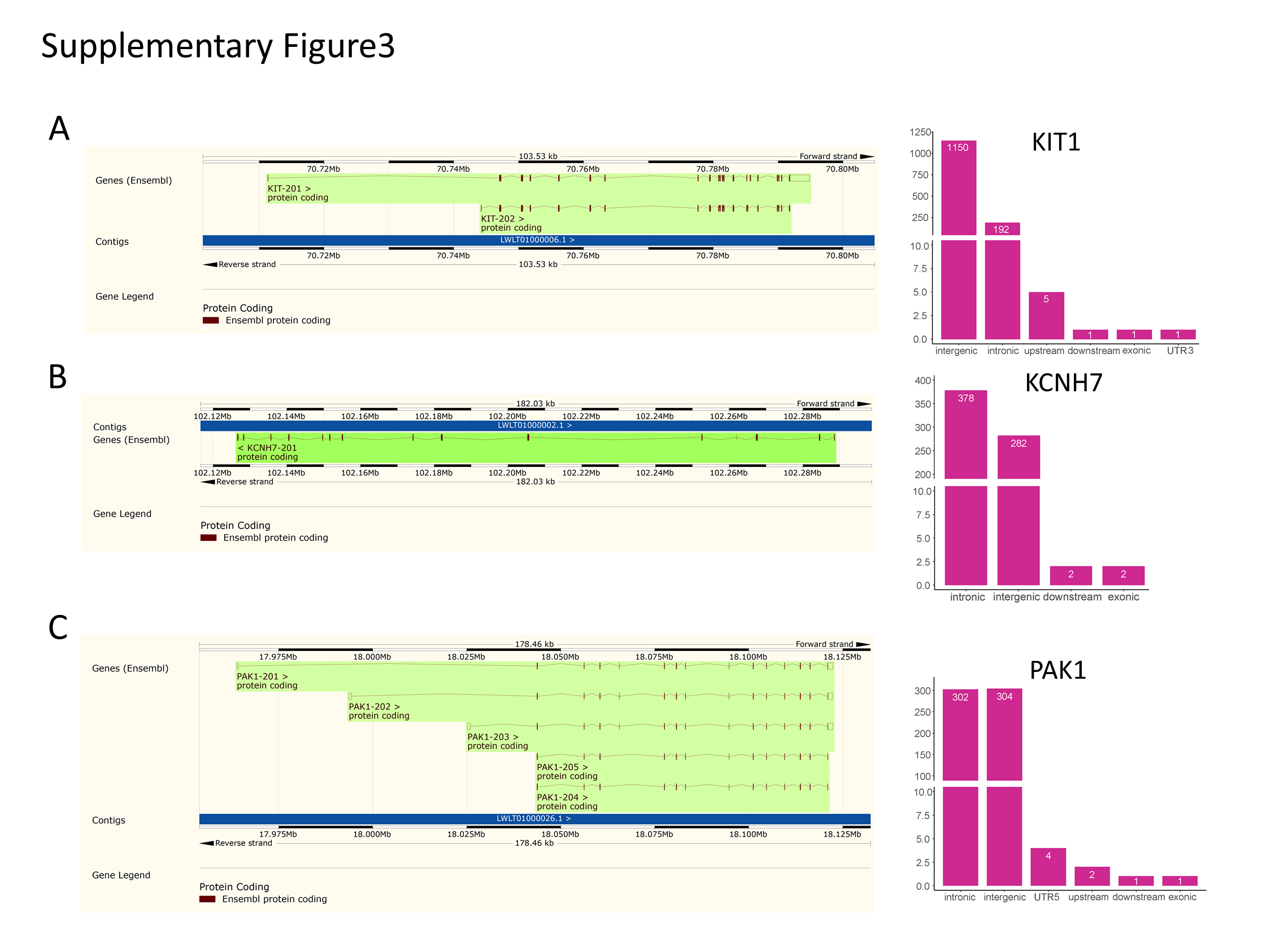

Supplement: FIGURE S3 — Structure of protein coding region of most candidate genes from Ensembl website. (A) Protein coding regions (left) and genomic mutation count (right) of KIT gene. (B) Protein coding regions (left) and genomic mutation count (right) of KCNH7 gene. (C) Protein coding regions (left) and genomic mutation count (right) of PAK1 gene. [file Image_3.TIF]
