# Supplementary material for: Genomic Signatures of Selection Associated With Litter Size Trait in Jining Gray Goat
Source: Front Genet. 2020 Mar 26;11:286. doi: 10.3389/fgene.2020.00286 (PMC7113370; doi:10.3389/fgene.2020.00286)
Supplement: TABLE S2 — Number of reads in quality control and mapping statistics. [file Table_2.DOCX]

Supplemental Table 2. Statistics of sequence reads in quality control and mapping.

| Sample | Raw Base (bp) | Clean Base (bp) | Effective Rate (%) | Q20 (%) | Q30 (%) | GC Content (%) | Mapped reads | Total reads | Mapping rate (%) |
| --- | --- | --- | --- | --- | --- | --- | --- | --- | --- |
| H-1 | 30517422300 | 30451083000 | 99.78 | 97.22 | 93.88 | 43.63 | 201574773 | 201953471 | 99.81% |
| H-2 | 34012028700 | 33915541800 | 99.72 | 96.92 | 95.33 | 44.29 | 223778763 | 226981091 | 98.59% |
| H-3 | 30574106400 | 30470269800 | 99.66 | 97.41 | 94.29 | 43.66 | 202044382 | 202376201 | 99.84% |
| H-4 | 40528495200 | 40409605500 | 99.71 | 97.09 | 95.56 | 44.23 | 267523845 | 270476849 | 98.91% |
| H-5 | 36776070600 | 36629977500 | 99.6 | 96.48 | 94.67 | 44.39 | 244908003 | 245506437 | 99.76% |
| H-6 | 39039855000 | 38832254700 | 99.47 | 97.05 | 95.52 | 44.03 | 260270757 | 260779608 | 99.80% |
| H-8 | 35101838100 | 34958247000 | 99.59 | 96.99 | 95.42 | 44.11 | 233888802 | 234378122 | 99.79% |
| H-9 | 39288218100 | 39186229200 | 99.74 | 97.2 | 95.73 | 44.08 | 260696016 | 262177834 | 99.43% |
| H-14 | 36627832200 | 36480535200 | 99.6 | 97.13 | 95.63 | 44.23 | 243278672 | 244528406 | 99.49% |
| H-15 | 41531475900 | 41343450000 | 99.55 | 96.97 | 95.4 | 44.16 | 276796830 | 277336093 | 99.81% |
| H-16 | 35250534000 | 35141071500 | 99.69 | 96.96 | 95.38 | 44.28 | 234779629 | 235317781 | 99.77% |
| H-17 | 39343848600 | 39174541800 | 99.57 | 96.81 | 95.16 | 44.12 | 261579108 | 262646900 | 99.59% |
| H-19 | 43798398300 | 43666883700 | 99.7 | 97.13 | 95.62 | 44.1 | 290110574 | 292286339 | 99.26% |
| H-20 | 37510259700 | 37399813800 | 99.71 | 97.05 | 95.52 | 44.15 | 249824155 | 250358789 | 99.79% |
| H-21B | 35797291800 | 35687011200 | 99.69 | 96.89 | 95.28 | 44.51 | 238486754 | 238948915 | 99.81% |
| H-22B | 35922519900 | 35770154400 | 99.58 | 96.94 | 95.35 | 44.14 | 239415539 | 239849367 | 99.82% |
| H-23B | 34633339200 | 34536779700 | 99.72 | 96.84 | 95.23 | 44.05 | 230651590 | 231147523 | 99.79% |
| H-24B | 36842397300 | 36721958100 | 99.67 | 97 | 95.44 | 44.25 | 245169184 | 245915114 | 99.70% |
| H-25B | 36606381600 | 36514782000 | 99.75 | 96.97 | 95.39 | 44.05 | 243266036 | 244270138 | 99.59% |
| H-26B | 34799213700 | 34597732500 | 99.42 | 97.02 | 95.47 | 44.12 | 231503990 | 232369110 | 99.63% |
| L-1 | 30272202900 | 30176399700 | 99.68 | 97.28 | 94.02 | 43.78 | 200012952 | 200400783 | 99.81% |
| L-2 | 30630165900 | 30480654900 | 99.51 | 96.91 | 93.47 | 43.68 | 201975524 | 202526342 | 99.73% |
| L-3 | 30253092000 | 30136872000 | 99.62 | 97.46 | 94.3 | 43.77 | 199908805 | 200382959 | 99.76% |
| L-4 | 31276128300 | 31158723300 | 99.62 | 97.18 | 94.02 | 43.72 | 206793647 | 207221066 | 99.79% |
| L-5 | 30778272300 | 30689785500 | 99.71 | 97.21 | 93.81 | 43.79 | 203213311 | 203530259 | 99.84% |
| L-6 | 36697523400 | 36498681300 | 99.46 | 96.93 | 95.36 | 44.25 | 244563915 | 245080746 | 99.79% |
| L-7 | 30721788600 | 30563255400 | 99.48 | 97.11 | 93.84 | 43.67 | 202872935 | 203304201 | 99.79% |
| L-8 | 32013503100 | 31886318400 | 99.6 | 97.49 | 94.16 | 43.64 | 211688747 | 211975117 | 99.86% |
| L-9 | 30147166200 | 30066351900 | 99.73 | 97.04 | 93.55 | 44.25 | 198972212 | 199285041 | 99.84% |
| L-10 | 31268211600 | 31129879800 | 99.56 | 96.91 | 93.47 | 43.72 | 206127225 | 206703837 | 99.72% |
| L-11 | 30952309500 | 30838115700 | 99.63 | 97.21 | 93.91 | 43.75 | 204111030 | 204836963 | 99.65% |
| L-12 | 30110018400 | 30031317900 | 99.74 | 97.4 | 94.21 | 43.52 | 198936278 | 199240627 | 99.85% |
| L-13 | 30662378700 | 30556378800 | 99.65 | 97.23 | 93.68 | 43.53 | 200858546 | 202671138 | 99.11% |
| L-15 | 30654794700 | 30591792000 | 99.79 | 97.31 | 93.97 | 43.55 | 202175756 | 202701589 | 99.74% |
| L-17 | 31142788800 | 31079868600 | 99.8 | 97.08 | 93.44 | 43.91 | 205099393 | 205754562 | 99.68% |
| L-18 | 30832140300 | 30759395100 | 99.76 | 97.5 | 94.17 | 43.58 | 203532311 | 204019127 | 99.76% |
| L-19 | 40201011900 | 40037442300 | 99.59 | 97.01 | 95.46 | 44.12 | 267504269 | 268389667 | 99.67% |
| L-20 | 31261079100 | 31084286100 | 99.43 | 97.08 | 94.14 | 43.94 | 206819201 | 207277567 | 99.78% |
| L-21B | 30246935400 | 30076384200 | 99.44 | 97.25 | 93.73 | 43.61 | 199588376 | 199939196 | 99.82% |
| L-22B | 32777384400 | 32674555200 | 99.69 | 97.3 | 94.22 | 43.71 | 216592398 | 217178464 | 99.73% |
